# Expression of Bovine Cytosolic 5′-Nucleotidase (cN-II) in Yeast: Nucleotide Pools Disturbance and Its Consequences on Growth and Homologous Recombination

**DOI:** 10.1371/journal.pone.0063914

**Published:** 2013-05-17

**Authors:** Simone Allegrini, Daniela Nicole Filoni, Alvaro Galli, Anita Collavoli, Rossana Pesi, Marcella Camici, Maria Grazia Tozzi

**Affiliations:** 1 Dipartimento di Chimica e Farmacia, Università di Sassari, Sassari, Italy; 2 Dipartimento di Biologia, Unità di Biochimica, Università di Pisa, Pisa, Italy; 3 Istituto di Fisiologia Clinica, Consiglio Nazionale delle Ricerche, Pisa, Italy; University of Minnesota, United States of America

## Abstract

Cytosolic 5′-nucleotidase II is a widespread IMP hydrolyzing enzyme, essential for cell vitality, whose role in nucleotide metabolism and cell function is still to be exactly determined. Cytosolic 5′-nucleotidase overexpression and silencing have both been demonstrated to be toxic for mammalian cultured cells. In order to ascertain the effect of enzyme expression on a well-known eukaryote simple model, we expressed cytosolic 5′-nucleotidase II in *Saccharomyces cerevisiae*, which normally hydrolyzes IMP through the action of a nucleotidase with distinct functional and structural features. Heterologous expression was successful. The yeast cells harbouring cytosolic 5′-nucleotidase II displayed a shorter duplication time and a significant modification of purine and pyrimidine derivatives concentration as compared with the control strain. Furthermore the capacity of homologous recombination in the presence of mutagenic compounds of yeast expressing cytosolic 5′-nucleotidase II was markedly impaired.

## Introduction

Cytosolic 5′-nucleotidase II (cN-II) is a widely expressed enzyme catalyzing the hydrolysis of phosphate esterified in 5′ position of nucleotides with a preference for 6 hydroxy-purine [1], [2]. The enzyme is involved in the regulation of IMP intracellular pool and therefore of purine nucleotides. The enzyme is expressed at higher level in cells and organs with high DNA turnover but it is present, although at low levels, also in organs without purine de novo synthesis and fixed cell population such as brain and heart [3], [4].

cN-II primary structure is highly conserved during evolution, in fact the identity between the zebra fish and the human enzyme is about 90% and between the bovine and the human enzyme is over 99% [5]. The analysis of the crystal structure of a truncated form of cN-II has shown that conserved regions, besides the active and regulatory sites, are present both in the protein core and in the interface between protein and solvent [6]. The widespread distribution and the extreme conservation of the protein sequence may suggest a fundamental role played by this enzyme in cell vital functions. In the past, the enzyme has been over-expressed (about 10 fold over the background) in human kidney embryonic cells (293T) causing a slight decrease of nucleoside and deoxynucleoside triphosphate pools (20–30%); the Authors however observed that the high cN-II expression was toxic for the 293T cells [7]. More recently, a constitutive silencing of cN-II has been obtained in a human astrocytoma cell line (ADF). In this case, cells undergo apoptosis as soon as cN-II specific activity falls below 40% as compared with the control [8]. Both these observations indicate that the level of cN-II must be kept constant. Despite substantial information on structural and functional characteristics available in the literature, the impact of the fluctuation of cN-II expression on nucleotide metabolism and in turn, on cell mechanisms regulated by nucleotides, has still to be elucidated. Human and bovine cN-II have been successfully expressed in *E. coli* [9], [10]; the expression of the enzyme in yeast, however, offers a number of advantages in comparison with prokaryotes such as the possibility to obtain a recombinant enzyme with some possible post-translational modifications. Furthermore, the recombinant yeast clone offers a good model for the study of the molecular consequences of cN-II expression in an eukaryotic organism, which is easy to grow and study. In *Saccharomyces cerevisiae* a soluble 5′-nucleotidase (Isn1p), coded by gene *ISN1*, has been described by Itoh et al [11], [12]. This protein shows an absolute specificity for IMP as substrate and a tetrameric structure similar to cN-II. Moreover, in silico sequence and structure analysis indicates that also the yeast nucleotidase belongs to the haloacid dehalogenase (HAD) superfamily, as already demonstrated for cN-II and other cytosolic 5′-nucleotidases [13], [14]. Nevertheless, the cN-II and the yeast enzyme differ for both substrate specificity and regulation. Moreover, the sequence alignment analysis revealed that Isn1p does not exhibit significant homology with any 5′-nucleotidase from other eukaryote or prokaryote organisms [12], [15]. In the present study we express bovine cN-II in yeast with the intent to study the impact of its expression on intracellular purine and pyrimidine compounds and on mechanisms strictly dependent on nucleotides such as cell growth and DNA repair. Our results demonstrate that the expression of cN-II in yeast causes a remarkable variation of ribonucleoside triphosphate and monophosphate pools, has a moderate effect on the duplication time, and reduces considerably the yeast ability to operate homologous recombination without affecting cell viability.

## Experimental Procedures

### Plasmid and Yeast Strain

The cN-II cDNA was amplified by PCR using pET-28c+cNII [10] as template. The forward primer (F_cNIIpYES2_EcoRI) is 5′-ATGGAATTCGCCACC**ATG**ACAACCTCCTG-3′. In bold the first triplet of cN-II ORF, boxed bases, spanning from −6 to +4, were designed to fit the Kozak consensus sequence [16] (http://en.wikipedia.org/wiki/Kozak_consensus_sequence). The reverse primer (R_cNIIpYES2_XhoI) is 5′-AGCTCCTCGAG**TCA**CTCCTCCTCTT-3′. Cycling parameters were: (1) 94°C for 2′; (2) 94°C for 45″; (3) 42°C for 30″; (4) 72°C for 4′; steps 2–4 repeated for a total of 4 cycles; (5) 94°C for 45″; (6) 57°C for 30″; (7) 72°C for 4′; steps 5–7 repeated for a total of 26 cycles (cycles at lower annealing temperature were necessary because at the beginning of PCR just about 50% of primers bind to template). PCR product was *EcoRI-XhoI* digested and cloned into the pYES2 plasmid (Life Technologies, Italy). In this vector cN-II ORF is placed under control of the inducible Gal1 promoter which allows the expression of the protein only when galactose is added to the growth medium. In this study, we used the diploid strain of *Saccharomyces cerevisiae* RS112 (*MAT* ***a/*** *MATα, ura3-52/ura3-52, leu2-3,112/leu2-Δ98, trp5-27/TRP5, ade2-40/ade2-101, ilv1-92/ilv1-92, arg4-3/ARG4, his3Δ5′-pRS6-his3Δ3′/his3-Δ200. LYS2/lys2-801*). Constructed by Robert Schiestl, it allows us to measure interchromosomal recombination events between the two *ade2* mutated alleles [17]. Complete media (YPAD), synthetic-complete (SC) and drop-out media (SC-) for yeast culturing were prepared according to standard procedures [18]. The RS112 strain was transformed with either pYES2 (control strain, from now on referred to as Y) or pYES-cNII plasmid (strain expressing cN-II, from now on referred to as N) according to the standard protocol [19]. Yeast cells carrying the plasmid were selected in plate containing medium lacking uracil (pYES2 contains *URA3* gene, missing in RS112 wild type cells). Single colonies were streaked again on selective medium and further analyzed.

### Expression of cN-II in *Saccharomyces Cerevisiae*

As mentioned before, in this system the expression of cN-II is driven by the galactose inducible promoter; therefore, single colonies of N strain were inoculated in 5 ml SC -Ura containing 2% glucose (SC Glu -Ura) and incubated for 24 hours at 30°C under constant shaking. The culture was centrifuged (6000×g for 20 min at 4°C), then the pellet was washed twice in sterile MilliQ water and split into two flasks: one containing 100 ml SC Glu -Ura and the other one 100 ml SC -Ura containing 5% galactose (SC Gal -Ura). The two cultures were incubated at 30°C for 24 hours under shaking. Thereafter, cells were washed twice with sterile MilliQ water and the pellets, collected by centrifugation at 6000×g for 20 min at 4°C, were either used immediately for protein expression analysis or stored at −80°C. As negative control, the same protocol was applied to Y strain.

### cN-II Purification

About 0.5 g of both Y and N strain pellets prepared as described in the previous section, were dissolved in 1 ml of 50 mM Tris HCl pH 8, 300 mM NaCl, 10 mM imidazole (lysis buffer) and cells were disrupted by vortexing the suspension in the presence of glass beds 0.5–1 mm of diameter, 10 times for 1 min each. The suspension was then centrifuged 30 min at 10000×g at 4°C. The supernatant was stored and the pellet subjected to the same treatment for a second time. The two resulting supernatants were combined and loaded (6 mg proteins) on a column packed with 1 ml of Perfect Pro Ni-NTA Agarose (5′Prime Inc, Gaithersbourg, MD 20878, US) resin and washed with lysis buffer (5 column volumes at 6 ml/h). The enzyme was eluted with a gradient 5 ml of lysis buffer +5 ml of the same buffer containing 100 mM imidazole, and fractions of approximately 200 µl were collected.

### Enzyme Assay

Phosphotransferase activity of cN-II was measured as the rate of [8-^14^C]IMP formation from 1.4 mM [8-^14^C]inosine, in the presence of 2 mM IMP (or GMP), 20 mM MgCl_2_, 4.5 mM ATP and 5 mM dithiothreitol, as previously described [20]. When required ATP was omitted or substituted by an equal concentration of 2,3-bisphosphoglyceric acid (BPG). One mU of enzyme activity represents the amount of enzyme required to convert 1 nmol of substrate to product per min under assay conditions.

### Protein Assay

The protein concentration was determined according to Bradford [21], using BSA as standard.

### Electrophoresis and Immunoblot Analysis

SDS-PAGE was performed following the method of Laemmli [22]. Yeast crude extract was mixed with SDS sample buffer (3% w/v SDS, 0.05% w/v Bromophenol Blue, 10% w/v glycerol, 1% v/v β-mercaptoethanol in 0.1 M Tris–HCl pH 6.8). Protein samples were applied to a 10% SDS-polyacrylamide gel. After electrophoresis, when needed, Western blot was performed using polyvinyl difluoride (PVDF) membrane and processed for immunodetection. Membrane was incubated with the cN-II antiserum (Sigma monoclonal Anti-NT5C2 Ab in mouse, Sigma-Aldrich, Milan, Italy) (1∶1000) overnight at 4°C. Detection of immunoreactive band was performed by using goat anti-mouse IgG horseradish peroxidase-conjugated (1∶5000) (Sigma-Aldrich, Milan, Italy) for 2 hours at room temperature. Visualisation was carried out using ECL kit reactives (Immobilon Western, Chemoluminescence, HRP Substrate, Millipore Corporation, Billerica, MA, USA).

### Cell Survival and Recombination Frequency

Both Y and N strains were routinely grown in SC Glu -Ura to be sure of the presence of the plasmid. From a stationary phase culture, aliquot containing 10^7^ cells were inoculated in 5 ml SC Gal -Ura in the presence of different concentrations of methyl methanesulfonate (MMS, Sigma-Aldrich, Milan, Italy), a DNA damaging agent that induces DNA breaks and increases homologous recombination in our system [17], [23]. Cells were incubated with MMS for 24 hours at 30°C under shaking. Then, cells were washed, counted and plated in duplicate as previously reported [17], [23]. Plates were incubated at 30°C for 2–4 days. Survival is expressed as number of colonies counted in the complete media divided by the number of cell counted (200). The frequency of interchromosomal recombination was expressed as number of ADE2 colonies/10^5^ vital cells. Data are reported as mean of three or more experiments.

### Growth Curve

The inocula to be used as starter for the growth curve experiments were prepared in 5 ml of SC Glu -Ura the day before, picking up one big yeast colony from plates (SC Glu -Ura agar medium) containing either Y or N strain. Cells were grown overnight at 30°C, under constant shaking, to an OD_600 nm_ of 2–3. Then the cells were washed twice in sterile MilliQ water to remove any trace of glucose and used to inoculate the new cultures (50 ml of SC Gal -Ura, distributed, after inoculation, in 15 sterile tubes each containing 3 ml). Initial densities were OD_600 nm_ = 0.01 when glucose was used, whereas OD_600 nm_ = 0.3 when galactose was the energy source of the medium. Cells were grown in the same conditions of the inocula. Cell growth was followed measuring OD_600 nm_. Experiments were run in triplicate. Doubling time was calculated as follows: 
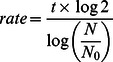
 were N and N_0_ are cell numbers (1 OD_600_ unit corresponds to 10^7^ cells/ml) at two different times in log phase of growth and t is the time in hours between N and N_0_.

### Temperature Resistance

N and Y yeast cells were grown for about 24 hours in SC Gal –Ura. Heat shock was performed in 1 ml of the liquid suspension for 2 min. at 60°C followed by immersion in ice cold water. Heat resistance of both strains was determined plating 500 µl of 1∶10^−3^ and 1∶10^−5^ dilutions of heat treated and untreated N and Y suspensions, on SC Gal –Ura agar medium and counting the colonies after 6 days of incubation.

### Intracellular Metabolite Extraction

To extract intracellular metabolites we used boiling ethanol as described by Canelas et al. [24] with some modifications. Aliquots of 7 ml (at least in triplicate) of Y and N strains were grown for 21.5 hours. At the desired time the samples were poured entirely into 21 ml of pure methanol (<5 sec), kept in a dry ice-ethanol bath (about −75°C), quickly vortexed and placed back into the ice-ethanol bath. The samples were centrifuged (3000×g for 5 min. at −20°C, with rotor pre-cooled at −20°C) and the supernatant discarded. Tubes containing 5 mL of 75% ethanol (v/v) were pre-heated in a water bath at 95°C for 5 min. Then, the boiling ethanol was quickly poured over the cell pellet of each sample (kept in the ice-ethanol bath); the mixture was immediately and quickly vortexed, and placed back in the water bath at 95°C. After 3 min, each tube was transferred back in ice-ethanol. Pellet was separated from supernatant by centrifugation (4000×g for 10 min at 4°C). The protein content of the pellets was determined using a modified Lowry method described by Peterson [25] whereas the supernatants were dried in a speed-vac (30°C for 6–8 hours) and resuspended in Milli-Q water (50–100 µL). Samples were stored at −20°C until needed.

### Extracellular Metabolite Extraction

500 µl of yeast suspensions (OD_600 nm_ was about 6 for Y strain and 10 for N strain) were centrifuged (15000×g, 1 min, at room temperature). The recovered supernatant was boiled for 5 min and centrifuged again (15000×g, 5 min, at room temperature). 200 µl of the supernatant were stored at −20°C. When these samples were analyzed for their base/nucleoside contents, the difference in the OD_600 nm_ of the initial suspensions was considered to normalize the obtained values.

### Capillary Electrophoresis Analysis of Nucleotides, Nucleosides and Bases

All the experiments were performed using either a Beckman P/ACE MDQ Capillary Electrophoresis System equipped with an UV detector or an Agilent Capillary Electrophoresis System equipped with a diode array detector.

The best resolution of the nucleoside-base contents either in culture media or in cell extracts was obtained with the following capillary electrophoresis conditions: uncoated silica capillary (75 µm id x 375 µm od; Polymicro Technologies, Phoenix, AZ, USA). The effective length of the capillary was 52 cm (60 cm total length). BGE was boric acid 125 mM, pH 8.5. The analyses were run at a constant voltage of 15 kV using a ramp time of 0.5 min. Temperature of the capillary was kept at 25°C. At the beginning of each working day the capillary was washed with water (1 min), NaOH 0.1 M (5 min) and water again (1 min); between runs with water (30 sec), NaOH 0.1 M (2 min), water again (30 sec) and equilibrated in BGE for 2 min.

To investigate the variation in nucleotide pool we applied some modifications to the protocol described by Friedecky et al. [26]. Briefly: uncoated silica capillary (75 µm id x 375 µm od; Polymicro Technologies, Phoenix, AZ, USA). The effective length of the capillary was 108 cm (115 cm total length). BGE buffer was citric acid 40 mM, titrated with GABA (γ-aminobutyric acid, powder, Sigma-Aldrich, Milan, Italy) at pH 4.4, containing 0.3 mM CTAB (hexadecyltrimethyl-ammonium bromide, Sigma-Aldrich, Milan, Italy). The analyses were run at a constant voltage of −30 kV using a ramp time of 0.5 min. Temperature of the capillary was kept at 25°C. At the beginning of each working day the capillary was washed with water (1 min), NaOH 0.1 M (5 min) and water again (1 min); between runs with water (1 min), NaOH 0.1 M (3 min), water again (1 min) and equilibrated in BGE for 2 min.

In both protocols all the solutions were syringe filtered (0.45 µm, Millipore Corporation, Billerica, MA, USA) before use. All samples were loaded by a low-pressure injection (for P/ACE equipment, 0.5 psi, 6 sec; for Applied instruments, at 34 mbar, 6 sec); these conditions ensured that the amount loaded was lower than 1% of the total capillary volume. The detection was performed either at 254 nm or 280 nm (in Applied instruments, spectrum of each peak was recorded).

### Statistical Analysis

All statistical analyses were performed using Student’s t test or the one-way analysis of variance (ANOVA) followed by Tukey’s multiple comparison test with the software InStat (ver. 3.05, GraphPad Software, Inc., La Jolla, CA 92037 USA).

## Results

### cN-II Expression and Purification in Yeast

**Figure 1:**
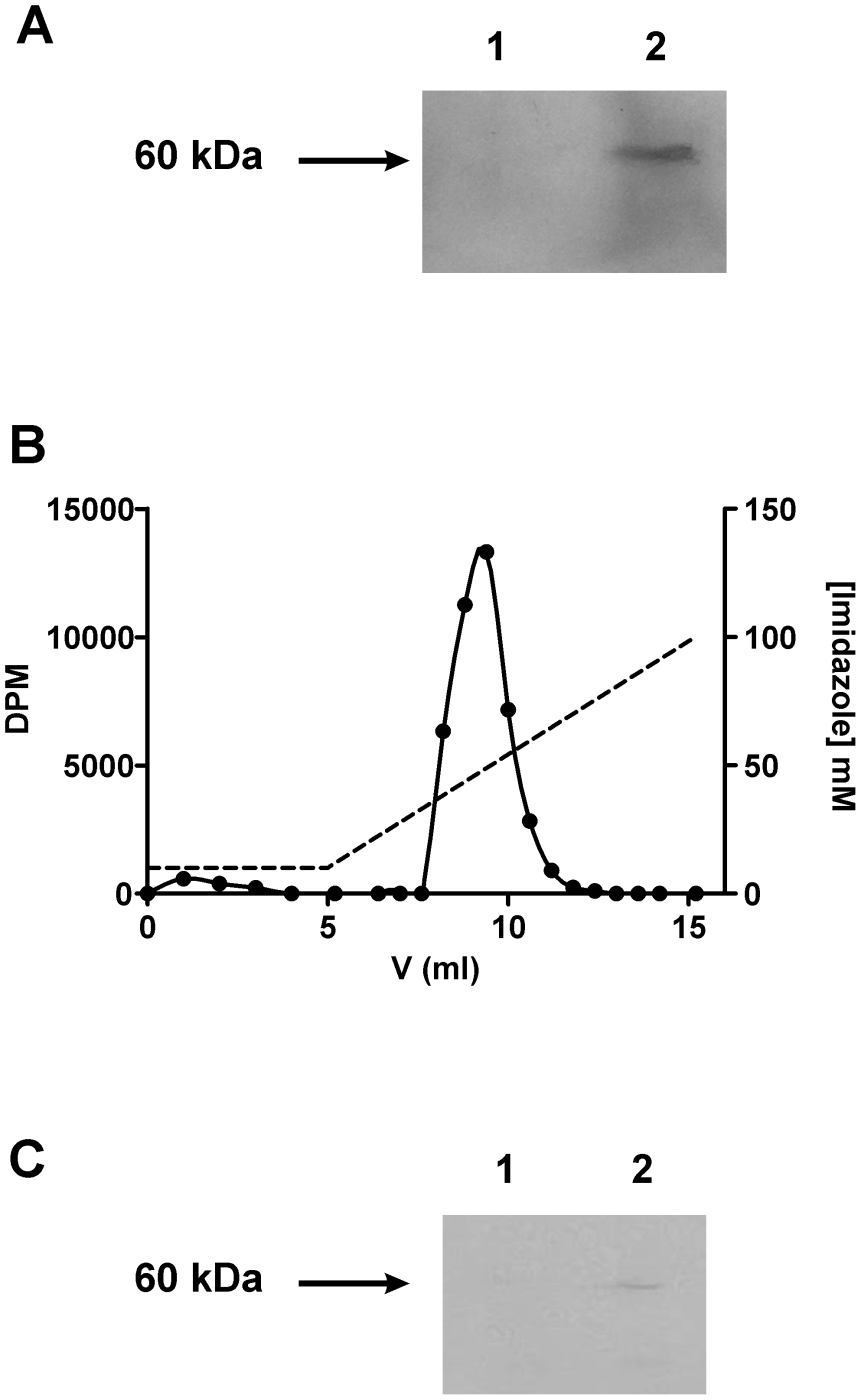
Purification and identification of cN-II in extracts of N yeast strain. A) Immunoblot analysis of Y and N strain crude extracts. Lane 1∶10 µg of protein of Y strain. Lane 2∶10 µg of protein of N strain. B) Elution profile of cN-II from N strain extract from IMAC resin. cN-II phosphotransferase activity is reported as DPM after 30 min of assay as described in Experimental Procedures (-•-). Dashed line (---) represents the imidazole gradient. Chromatography conditions were as described in Experimental Procedures. Y strain extracts, treated in the same way, showed no phosphotransferase activity in the same fractions (data not shown). C) SDS-PAGE of purified (as described in B) N and Y strain extracts. Lanes are as described in panel A. In this case 5 µg of proteins were loaded (lane 1 and 2).

Crude extracts from *Saccharomyces cerevisiae* RS112 strain expressing cN-II (N) and control cells (Y) were assayed for phosphotransferase activity with either GMP or IMP as substrate. Only a couple of soluble 5′-nucleotidases belonging to HAD superfamily possess the phosphotransferase activity [14], [27] and this has been utilized as a cN-II specific assay with very low interference by other enzyme activities, while the ability to catalyze the hydrolysis of nucleoside monophosphates is shared among a number of specific or aspecific hydrolases [28]. Table 1 shows that the phosphotransferase activity expressed by Y strain in the presence of GMP as phosphate donor and inosine as acceptor is approximately four times lower than the activity measured with IMP (1.1 vs 4.3 mU/mg). A previous report indicated that a nucleotidase acting exclusively on IMP is expressed in yeast but no attempt was made to ascertain if this nucleotidase possessed a phosphotransferase activity as well [11]. N strain, however, showed a phosphotransferase specific activity considerably higher than Y strain (5 and 13 fold with IMP and GMP as phosphate donor, respectively), with very little difference between the two substrates. The phosphotransferase activity of cN-II in N strain is activated by the effectors ATP and BPG (Table 2) and has never been described in yeast [11]. Immunoblot analysis with antibodies specifically raised against bovine cN-II indicates that this enzyme is present in the crude extract of N yeast cells but not of Y cells (Figure 1A). We applied the Ni-NTA Agarose resin purification protocol to both Y and N strain crude extracts. This IMAC pseudo-affinity chromatography has been shown to give a high purification factor since cN-II is peculiarly rich in histidine residues and firmly binds the metal even if expressed without His-tag [9]. The chromatographic elution profile of the N strain extract exhibited a peak of phosphotransferase activity, eluted with the imidazole gradient, which was absent with the Y strain extract. As expected, the electrophoretic analysis of the pooled, active fractions indicates the presence of a 60 kDa protein band (Figure 1C) only in the N strain purified extract, with a purification factor of approximately 100 fold.

**Table 1:** Phosphotransferase activity (mU/mg) in crude extracts of Y and N strain with different phosphate donors.

	GMP	IMP
Y	1.1±0.04	4.3±0.08
N	14.6±0.1	16.5±0.1

N represents the strain expressing cN-II whereas Y the control strain. Assay was performed as described in Experimental Procedures. Values are the mean±SD of at least three independent samples.

**Table 2:** Effects of activators on phosphotransferase activity in crude extracts of Y and N strains.

	No effectors	+ATP	+BPG
Y	3.47±0.01	3.46±0.01	3.55±0.02
N	3.56±0.02	15.07±0.09	15.41±0.09

Activity is expressed as mU/mg. N represents the strain expressing cN-II whereas Y the control strain. Assay was performed as described in Experimental Procedures. Values are the mean±SD of at least three independent samples.

### Effect of cN-II Expression on Yeast Growth

**Figure 2:**
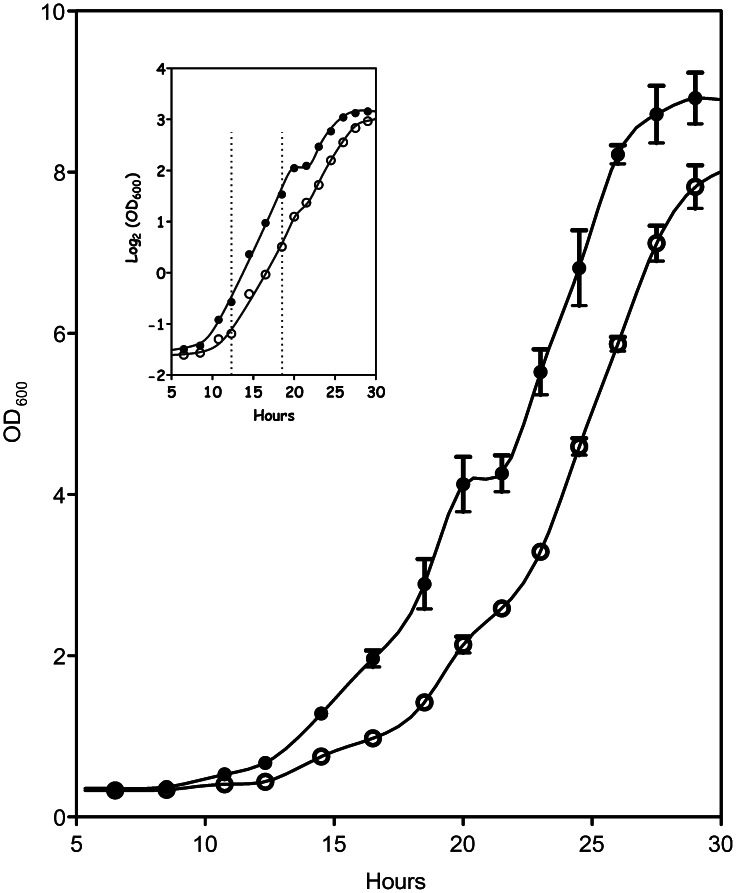
Effect of cN-II on yeast growth with galactose. Growth curve of N (-•-) and Y ( -○- ) strain in the presence of 5% galactose in the culture medium. Cell growth was followed as described in Experimental Procedures. The logarithmic growth period (between the two dotted lines) that we used to calculate the duplication times of the two strains is indicated in the inset. Error bars represent SD of the mean of three independent determinations.

Since the transcription of cN-II in our plasmid is under gal-promoter control, to express cN-II we used a medium containing 5% galactose. Under these growth conditions, yeast cells ferment galactose to ethanol; consequently, the decrease of sugar concentration causes *Saccharomyces cerevisiae* to undergo a transition termed diauxic shift. During this time, cell growth is transiently arrested and the cell metabolism is shifted from fermentative to oxidative metabolism. In this second phase, the doubling time is considerably higher than during fermentative growth [29]. Figure 2 shows that during the first (fermentative) phase of the growth N strain grows faster than Y (doubling times, calculated during the logarithmic growth - see inset - are 2.9 hours and 3.6 hours respectively). The same experiment performed in the presence of glucose, a repressor of the expression of galactose regulated genes, and therefore in the absence of cN-II, results in superimposable growth curves for both strains (results not shown).

### Effect of cN-II Expression on Purine and Pyrimidine Metabolite Pools

In order to better understand the influence of cN-II activity on intracellular nucleotide composition, we extracted these compounds from both Y and N strain after 21.5 hours of growth. The capillary electrophoretic method described in Experimental procedures can discriminate between ribo and deoxyriboderivatives [26], but, due to their very low intracellular concentration, deoxynucleotides are almost undetectable in our experimental conditions, therefore only ribonucleotides and nucleosides are reported. Table 3 shows the intracellular nucleotide and nucleoside distribution in N strain with respect to Y strain. Our results indicate that N strain showed significant variations of both nucleoside tri- and monophosphate pools, whereas no relevant differences were measured in nucleoside diphosphate pools. Nevertheless the sum of adenine nucleotides (AMP+ADP+ATP) and IMP did not decrease in N strain when compared with Y strain, indicating that the accumulation of AMP and IMP fully accounts for the apparent loss of ATP. The same observation can be made for the guanylic compounds. Surprisingly, inosine and hypoxanthine content in N strain is significantly lower than in Y strain. This last result is confirmed by the observation that, at the end of the log phase, just 60% of hypoxanthine and 35% of inosine are present in the N strain growth medium with respect to the Y strain. (data not shown).

**Table 3:** Pools of nucleosides and nucleotides in yeast cell extracts.

	N^a^	Y^a^	statistics
UTP	41±16	136±49	p = 0.033
GTP	41±14	123±45	p = 0.04
ATP	374±46	1175±300	p = 0.01
CTP	52±20	214±87	p = 0.03
UDP	66±19	93±48	NS
GDP	53±10	39±10	NS
ADP	441±66	304±133	NS
CDP	53±13	84±20	NS
UMP	115±28	nd	
GMP	77±5	nd	
AMP	384±109	86±42	p = 0.012
IMP	62±10	nd	
Inosine	78±9	362±17	p<0.0001
Ipoxanthine	176±47	399±12	p<0.0001
UDPG	343±39	571±178	NS
NAD	1374±313	1494±374	NS
NADP	67±27	40±10	NS
AXP+IMP	1337±212	1590±524	NS
GXP	172±28	186±52	NS
AMP/ATP	1.1±0.14	0.0955±0.0096	p = 0.0098
EC	0.537±0.081	0.83±0.036	p = 0.0047

Shadowed are the compounds in which a significant variation was measured between N and Y strain. Values are the mean± SD of at least three independent samples, and are expressed in arbitrary units. Each value is calculated dividing the peak area in the electropherogram by the value of protein concentration of the pellet of the same sample (see Experimental Procedures).

nd = not detectable; NS = not significant; UDPG = UDP-glucose; AXP = ATP+ADP+AMP; GXP = GTP+GDP+GMP; EC = energy charge.

a N represents the strain expressing cN-II whereas Y the control strain.

These results demonstrate that overexpression of cN-II in yeast cells does not lead to a net decrease of the purine ring content. On the other hand, it causes a consistent increase of ADP/ATP (about 4 fold) and AMP/ATP (about 11 fold) ratios which regulate nutrient metabolism in many organisms [30]. Furthermore, AMP accumulation may also have an important impact on signalling pathways. In particular, the *Saccharomyces cerevisiae* AMP kinase homolog, Snf1, activated by energy depletion, is involved at different levels in the regulation of many cellular processes including glycogen storage and thermal resistance [31]. N strain was 100% resistant to 2 min. exposure to 60°C, while just 60% of Y strain cells survived at the same treatment.

### Effect of cN-II Expression on Homologous Recombination

**Figure 3:**
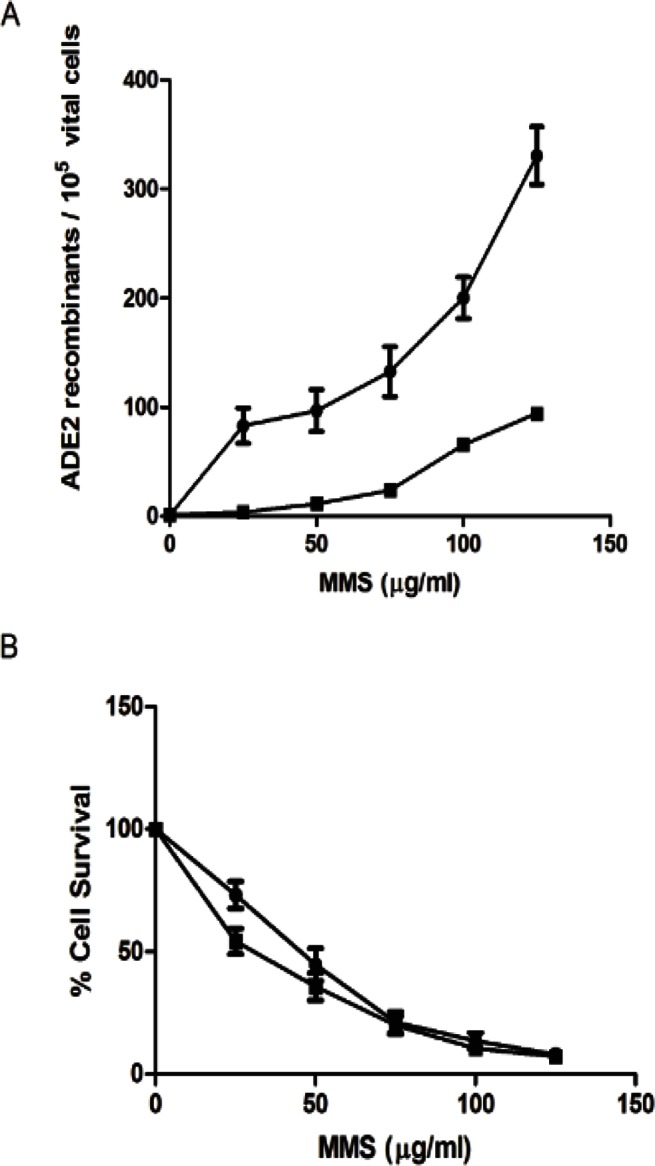
Effects of cN-II expression on recombination and survival of yeast. The espression of cN-II in yeast culture incubated in the presence of increasing concentration of MMS, affects their ability to operate recombination (A) with no evident influence on their survival response to the DNA damaging agent (B). Y strain (-•-) and N strain (-▪-). Error bars represent SD of the mean of three independent determinations.

The analysis of intracellular nucleotide content indicates a substantial impact of cN-II on purine and pyrimidine compounds. Therefore we decided to investigate whether this unbalance in nucleotide and nucleoside pools could be detrimental for the cells. In fact, we measured both cell viability and recombination frequency in N and Y strains treated with the mutagenic compound MMS. As reported in the Figure 3B no evident difference between the two strains is observed in the cell survival as a function of MMS concentration. On the contrary in N strain we observed a remarkable decrease in the recombinant events as a function of increasing MMS concentration when compared to Y strain. Very preliminary results were presented in an international meeting and reported in the proceedings [32]. Here we show consolidated results suggesting that the cN-II expression in *Saccharomyces cerevisiae* reflects on the yeast ability to repair DNA damage via homologous recombination without affecting the survival.

## Discussion

**Figure 4:**
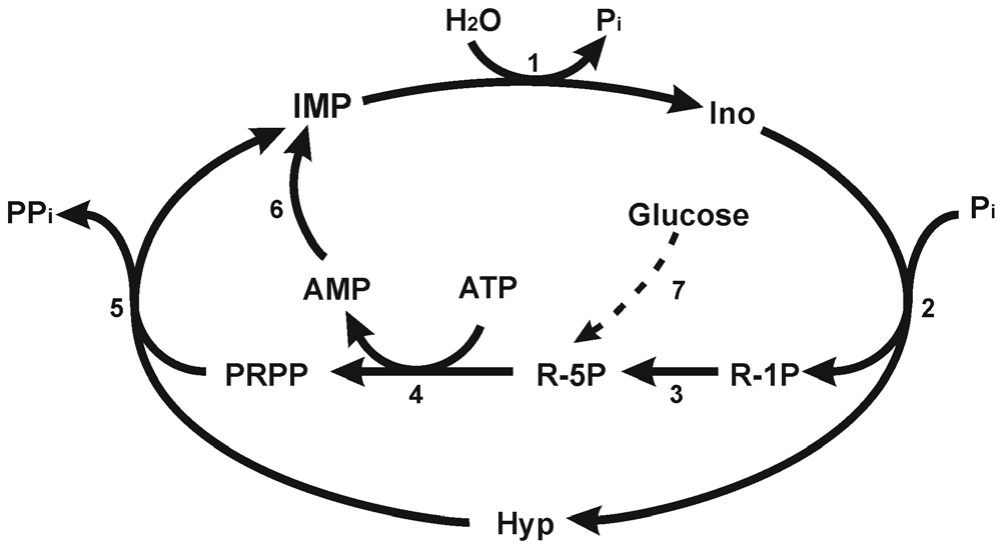
Scheme of the regulation of intracellular IMP levels. 1) soluble 5′-nucleotidases (Isn1p and, in N strain, mainly cN-II); 2) purine nucleoside phosphorylase; 3) phosphoribomutase; 4) PRPP synthase; 5) hypoxanthine-guanine phosphoribosyl transferase; 6) AMP deaminase; 7) pentose phosphate pathway.

The structure and catalytic mechanism of cN-II have been extensively studied but many aspects of its physiological role are still obscure. Because of its substrate specificity, it is obvious that the enzyme is involved in the regulation of intracellular IMP concentration; in fact, Ipata et al. proposed that the enzyme is participating in a cycle regulating intracellular IMP and phosphoribosyl pyrophosphate (PRPP) concentrations, also determining the destiny of newly synthesized or salvaged IMP, mediating hypoxanthine or uric acid production and nucleotide interconversion [5], [33] (see Figure 4). cN-II is the rate limiting step of the cycle; depending on enzyme expression, energy charge, diadenosine tetraphosphate (Ap_4_A), inorganic phosphate (Pi) and IMP concentration, the cycle may have different rate. It must be noted that a complete cycle attains a net conversion of ATP into AMP. However, whether the rate of the cycle and of cN-II activity affects a number of fundamental cell mechanisms, is still to be determined. To address this issue, our experimental approach was to silence the enzyme in a human cell model [8] and, as reported in this paper, to obtain the heterologous expression of cN-II in yeast, in order to track its impact on several cell functions. In fact, purine and pyrimidine compounds are of paramount importance in the metabolic regulation and the rate of their catabolism, salvage and interconversion is determinant for the rate of carbohydrate catabolism and for the respiro-fermentative transition [34]. Yeast expresses all the enzymes involved in the cycle described in Figure 4, but IMP hydrolysis depends on Isn1p, a nucleotidase acting only on IMP [11] expressed at a very low level, and not modulated by energy charge. By the transformation of RS112 yeast strain with pYES2 carrying the ORF of bovine cN-II we obtained a new yeast strain in which the 5′-nucleotidase acting on IMP and GMP was regulated as the vertebrate enzyme and was considerably higher than in the wild type. Since the enzyme is involved in purine catabolism it is conceivable that its expression may impact on intracellular purine compounds. Our results indicate that a decrease of purine and pyrimidine nucleoside triphosphates and a strong increase of AMP/ATP and ADP/ATP ratios are the major effects exerted by cN-II expression. With our analytical method we were unable to determine the deoxynucleotide and deoxynucleoside distribution in our yeast strains, but the observation that N strain grows faster than Y strain suggests that deoxynucleotide pools are not heavily affected by cN-II expression. Furthermore, intracellular concentration of nucleoside diphosphates, substrates of ribonucleotide reductase, were unaffected by cN-II expression. A cN-II overexpression was described in 293T cultured cells [7], [35]. The Authors observed that a cN-II level about 10–30 fold higher than control cells caused a modest decrease of about 20–30% of purine and pyrimidine nucleoside triphosphates, followed by a significant increase of duplication time, which, on the other hand, was not proportional to the degree of cN-II overexpression. The Authors formulated the hypothesis that the decrease in growth rate was not directly related to both nucleotidase activity and nucleoside triphosphate depletion [7], [35].

cN-II represents a regulated step in the cycle reported in Figure 4. The cycle causes ATP consumption due to the PRPP synthesis. If the rate of the cycle is high, the ATP breakdown may be faster than the rate of ADP phosphorylation, generating an AMP increase due to adenylate kinase activity. AMP is deaminated to IMP which, in turn, may be converted into GMP. Therefore, in N strain, cN-II causes a significant increase of the ratios ADP/ATP and AMP/ATP that regulate many cell functions including activation of Gal operon through Snf1p activation [36]. In this respect, N strain of *Saccharomyces cerevisiae* represents a model in which the ratio AMP/ATP and the intracellular nucleotide concentration are chronically altered. Snf1p activation, stimulated by energy depletion, is required for the modulation of a number of protein functions and the transcription of a large number of genes and in particular those needed for the transport and metabolism of fermentable carbon sources alternative to glucose [36]. It is therefore conceivable that N strain is faster in removing the catabolite repression and in activating gal operon. Furthermore Snf1p is involved in the cellular responses to several environmental stresses such as heat shock [31]. Indeed, N strain resulted to be more heat resistant than Y strain. Taking together, our results indicate that cN-II expression *in Saccharomyces cerevisiae* causes ATP depletion and AMP accumulation. This alteration may cause a stimulation of Snf1p activity which in turn enhances galactose catabolism. On the other hand, the depletion of nucleoside triphosphates exerts adverse effects on fundamental cell mechanisms and in particular on the recombination rate. The homologous recombination is one of the mechanisms involved in DNA repair [37]. In the absence of mutagenic compounds the recombination frequency of N and Y strains is similar. However in stressing conditions, generated by MMS, N strain shows a lower recombination frequency compared to Y strain. This occurrence might well be ascribed to the depletion of ATP, which is strongly required in all DNA repair mechanisms, but more complex mechanisms, directly or indirectly related to cN-II, cannot be ruled out. Work is in progress to ascertain if cN-II can interfere with DNA damage checkpoint or downstream mechanisms involved in homologous recombination.
